# Genital arousal and responsive desire among women with and without sexual interest/arousal disorder symptoms

**DOI:** 10.1093/jsxmed/qdae036

**Published:** 2024-04-06

**Authors:** Shari M Blumenstock, Kelly Suschinsky, Lori A Brotto, Meredith L Chivers

**Affiliations:** Department of Psychology, Queen’s University, Kingston K7L 3L3, Canada; Kinsey Institute, Indiana University–Bloomington, Bloomington IN 47405, United States; Royal Ottawa Mental Health Centre, University of Ottawa, Ottawa K1Z 7K4, Canada; Department of Obstetrics and Gynaecology, University of British Columbia, Vancouver V6T 2A1, Canada; Department of Psychology, Queen’s University, Kingston K7L 3L3, Canada

**Keywords:** relationship quality, sexual arousal, sexual desire, sexual dysfunction, sexual psychophysiology, thermal imaging, photoplethysmography

## Abstract

**Background:**

Models depicting sexual desire as responsive to sexual arousal may be particularly apt for women experiencing arousal or desire difficulties, and the degree to which arousal triggers desire may depend on the relationship context and desire target and timing—yet, these associations have not been directly tested among women with and without sexual interest/arousal disorder (SIAD).

**Aim:**

To assess the role of SIAD status and relationship satisfaction in the associations between genital arousal and 4 types of responsive desire.

**Methods:**

One hundred women (*n* = 27 meeting diagnostic criteria for SIAD) in romantic relationships with men viewed a sexual film (pleasurable intimate depiction of oral sex and penile-vaginal intercourse) while their genital arousal was recorded via vaginal photoplethysmography (*n* = 63) or thermal imaging of the labia (*n* = 37). Partner and solitary desire was assessed immediately before and after the film (immediate desire) and 3 days later (delayed desire).

**Outcomes:**

Outcomes consisted of genital response (*z* scored by method) and associations between genital response and responsive sexual desire.

**Results:**

The key difference between women with and without SIAD was not in their ability to experience genital arousal but in how their genital responses translated to responsive sexual desire. Women with SIAD actually exhibited greater genital arousal than unaffected women. Associations between genital arousal and desire were significant only for women with SIAD and depended on relationship satisfaction and desire type. For women with SIAD with low relationship satisfaction, higher arousal predicted lower immediate desire for a partner; for those with high relationship satisfaction, arousal was either positively related (vaginal photoplethysmography) or unrelated (thermal imaging of the labia) to immediate desire for a partner. Associations with other desire types were not significant.

**Clinical Implications:**

Patterns of genital arousal and partner-specific responsive desire among women affected with SIAD were indicative of an avoidance model in response to heightened genital arousal, unless relationship satisfaction was high; attending to genital arousal sensations could be a means of triggering sexual desire for women with SIAD who are satisfied in their relationships.

**Strengths and Limitations:**

This is one of the first sexual psychophysiologic studies to connect relationship factors to patterns of sexual response. The differing arousal assessment procedures and lack of official diagnosis may have attenuated results. The homogeneous sample and in-person session requirement limit generalizability.

**Conclusion:**

When compared with unaffected women, women affected by SIAD may exhibit stronger arousal responses with sufficiently incentivized sexual stimuli, and the connection between their genital arousal and responsive desire for their partners may be stronger and more dependent on relationship context.

Contemporary theoretical models of sexual response indicate that sexual desire emerges from arousal or is responsive to sexual stimuli.^1^^,^^2^ The incentive motivation model posits that the body responds to the experience of physiologic arousal by evaluating sexual stimuli and motivating sexual behavior, and this connection is influenced by additional personal and external factors.^1^^,^^2^ This model of responsive desire, while applicable to many individuals, may be particularly apt for women who experience sexual difficulties.^3^ Sexual interest/arousal disorder (SIAD) is a clinical condition characterized by a persistent diminished ability to become sexually aroused or experience sexual desire,^4^^,^^5^ and it affects as many as 1 in 3 women.^6^ Despite the known detriments to well-being, the underlying patterns of arousal and desire for women with and without SIAD are not well understood. Documenting these patterns and other moderating factors represents an important step in identifying potential routes for treatment.

Studies comparing genital responses to sexual stimuli (via vaginal photoplethysmograph [VPP]) among women with and without sexual difficulties have indicated mixed findings. Some report no differences in genital arousal.^7–9^ Others note lower genital arousal among women with sexual arousal dysfunctions as compared with women who have general sexual dysfunctions or are sexually functional,^10^ or they find reduced genital response among women with genital arousal difficulties vs healthy controls but no differences when comparing controls with other arousal disorders (subjective and combined subjective and genital).^11^ Our first aim (research aim 1) was to add to this literature by assessing genital response to sexual stimuli among women with and without SIAD-specific symptoms. Furthermore, in light of the mixed findings in arousal responses to sexual stimuli among women with and without sexual difficulties, it remains unclear how arousal is linked to desire among women with SIAD and whether the links differ from women without SIAD. The second aim (research aim 2) was to assess the role of SIAD status in the connections between genital arousal and responsive desire.

A key feature of the incentive motivation model is that the connection between arousal and desire depends on other relevant factors. Notably, romantic relationships are a common context in which women experience distressing sexual difficulties, such as low desire,^12^^,^^13^ and relationship factors have been linked to several sexual outcomes within romantic relationships, including desire.^3^^,^^13–15^ The incentive motivation model emphasizes that the emergence of desire from arousal is partially determined by the incentive value placed on a sexual stimulus and the sexual experiences that it represents.^2^ Emotional connection with a romantic partner plays an influential role in motivating sexual activity with that partner,^16^^,^^17^ suggesting that the quality of a romantic relationship could affect the value of sex with a romantic partner and, in turn, the degree to which genital arousal triggers desire for that partner. Recent evidence indicates the powerful role that relationship satisfaction plays in links between subjective arousal and responsive desire, with high relationship satisfaction enhancing the ability of subjective arousal to trigger desire for a current partner.^18^ Moreover, recent advances in the study of sexual desire have highlighted the crucial need to distinguish among desire targets when investigating what predicts sexual desire.^19^^,^^20^ Desire measures commonly used today typically distinguish between solitary and dyadic desire.^21^^,^^22^ Much of the literature on women and low desire has focused on women in romantic relationships and, often implicitly, sexual desire specifically for their current romantic partners. Solitary sexual activity likely represents a very different sexual experience as compared with sex with a romantic partner. Timing presents an additional dimension: desire can be experienced immediately after exposure to an incentivized sexual stimulus, yet desire has also been found to sustain beyond initial exposure (eg, 24 hours).^23^ Models of responsive desire do not specify how long desire lasts once triggered, and this has not been systematically tested. Differentiating between immediate and delayed desire could offer additional insight into sexual response patterns and the course of responsive desire. For women with SIAD, it is possible that their desire may not last as long after experiences of arousal than women without SIAD, which would result in different patterns for immediate vs more delayed desire. As such, along with relationship satisfaction, desire type represents an additional potential moderator for the link between arousal and desire. Previous evidence has shown that relationship satisfaction modulates the connections between subjective arousal and various types of desire,^18^ but the roles of relationship quality and desire type are unknown for patterns involving genital arousal. Thus, our third aim (research aim 3) was to assess whether the degree to which genital arousal triggers desire depends on relationship satisfaction and desire type (ie, partner vs solitary targets; immediate vs delayed timing).

## Methods

### Participants and procedure

Data are drawn from 2 studies designed to assess the role of SIAD symptomology in sexual response patterns. Both studies involved women attending laboratory sessions during which their genital response was assessed while viewing an 11-minute sexual film, which portrayed an attractive heterosexual couple engaging in sexual activities (cunnilingus, fellatio, and penile-vaginal intercourse) that was depicted as affectionate and mutually pleasurable.

Responsive sexual desire was assessed twice: immediately after the film and 3 days later. Genital arousal was assessed via VPP in the first study and thermal imaging of the labia (TIL) in the second. The first affiliated research ethics board approved all procedures; written informed consent was provided by all participants. More detailed information about the procedures and exclusion criteria in each study can be found in Blumenstock et al.^17^

Both studies recruited women aged 18 to 50 years currently in sexual or romantic relationships with men: 77 in the VPP study and 47 in the TIL study. Data from 24 participants were excluded due to poor data quality or being in nonromantic sexual relationships with men. The final sample for the current analyses included 100 women, with 27 meeting diagnostic criteria for SIAD.^4^

### Measures

#### SIAD status

To assess SIAD status, a 6-item screening tool^24^ was administered. A probable SIAD diagnosis was made if participants endorsed ≥3 of the 6 criteria (each lasting at least 6 months and on the majority of sexual encounters) and significant personal distress. The 6 criteria included a lack of interest in sexual activity and/or lack of responsive desire; reduced initiating sex or being receptive to sexual advances; and reduced or absent erotic thoughts, sexual pleasure, or sexual sensations.

#### Genital sexual arousal and data reduction

##### Vaginal photoplethysmograph

The VPP assessed changes in vaginal pulse amplitude sampled at a rate of 200 Hz, bandpass filtered at 0.5 to 10 Hz, and digitized at 40 Hz (TechnischeHandelsondernemingCoos). Data were recorded via a BIOPAC system (BIOPAC Systems Inc). Vaginal pulse amplitude is a measure of genital vasocongestion and is considered a valid measure of genital sexual response associated with sexual arousal.^25^

##### Thermal imaging of the labia

The thermal imaging camera ﻿(TS9230 Thermo Tracer; NEC Avio Co, Soltec Inc) had 0.08 °C sensitivity and continually sampled women’s genital temperature at a rate of 60 Hz, averaged to 1 frame per second. The region of interest was the left labia majora.^26^^,^^27^ Evidence for convergent and discriminant validity of thermography, particularly with labial temperature, has been documented.^26^^,^^28^

##### Data reduction and z scores

Movement artifacts in the vaginal pulse amplitude waveforms were identified via visual inspection and deleted.^29^ For the thermography data, an automated region-of-interest tracking procedure was used.^27^ For each study group, mean genital response was computed across 30-second bins during the film^30^; genital response relative to baseline was computed by subtracting the baseline score (average response for 20 seconds prior to the sexual film) from the mean. *Z* scores were then used to create a combined measure with genital responses from both study groups (calculated separately by study group). *Z* scores standardize the distribution of a variable in a particular sample, thus facilitating the comparison between 2 samples with different distributions.

#### Responsive sexual desire

Sexual desire was assessed immediately prior to the film (prestimulus baseline), immediately following it (immediate desire), and 3 days later (delayed desire). For immediate desire, items assessed partner desire (“How strong is your desire for sex with a partner?”) and solitary desire (“How strong is your desire to masturbate?”); the response scale ranged from 0 to 9 for both items. These items have been used extensively in prior research,^18^^,^^31^ and single items minimized participant fatigue during the laboratory visit.

To assess delayed desire, the validated multi-item Report of Behaviours and Feelings–Desire^32^ was administered in the 3-day follow-up questionnaire. Prompts asked participants to indicate their frequency of certain behaviors, thoughts, or feelings in the past 3 days, with a response scale from 0 (not at all) to 5 (≥5 times). Desire for a current romantic partner (delayed partner desire) included 3 items (eg, fantasized about sex with a current partner). Desire to masturbate or behave sexually alone (delayed solitary desire) included 3 items assessing masturbation or use of erotica. Items were summed where higher values equated to higher desire (Cronbach’s alpha = 0.908 for partner desire and 0.606 for solitary desire).

Baseline 3-day desire was also assessed with the Report of Behaviours and Feelings–Desire in the baseline questionnaires, with the prompt asking about a “typical 3-day period.”

#### Relationship satisfaction

The Relationship Assessment Scale^33^ includes 7 items (eg, “In general, how satisfied are you with your relationship?”) and response scales from 1 (eg, low satisfaction) to 5 (eg, high satisfaction). Items were reverse scored when applicable and summed, with higher values equating to higher relationship satisfaction (Cronbach’s alpha = 0.849).

#### Gender attraction

A modified Kinsey Sexual Attraction Scale assessed gendered sexual attractions. The response scale ranged from 0 (sexually attracted to men only) to 6 (sexually attracted to women only). Responses were coded as 1 (sexually attracted to men only) and 0 (other responses).

### Data analyses

Data were analyzed via the R environment^34^; visualizations were created via the *ggplot2* package.^35^ Linear regression models assessed the following:

1) SIAD status as a predictor of genital arousal (research aim 1)
2) the interaction between SIAD status and genital arousal as predictors of the 4 desire types (research aim 2)
3) the interactions among SIAD status, genital arousal, and relationship satisfaction as predictors of the 4 desire types (research aim 3)

To assess whether the associations differed by study group, interactions with the study group were entered first. If there were no significant interactions with the study group, the interactions were removed from the model.

## Results

Table 1 presents sample characteristics. Descriptive statistics of key variables and group comparison test results are presented in Table 2. Preliminary results from *t*-tests indicated that for those in the VPP group, women with SIAD reported lower immediate partner desire, delayed partner desire, and baseline 3-day desire as compared with unaffected women. For those in the TIL study group, women with SIAD reported lower levels of all desire types, including baseline levels, with the exception of delayed solitary desire.

**Table 1:** Demographic characteristics of the sample and group differences across SIAD status.

	**SIAD status**							
	**Non-SIAD**		**SIAD** ^a^				**Total**	
**Characteristic**	**No.**	**%**	**No.**	**%**	**χ** ^**2**^	***P* value**	**No.**	**%**
Total	73	100	27	100			100	100
Study group					0.88	.348		
VPP	48	65.8	15	55.6			63	63.0
TIL	25	34.2	12	44.4			37	37.0
Race/ethnicity					5.97	.202		
Asian	9	12.3	2	7.4			11	11.0
European	49	67.1	22	81.5			71	71.0
Other^b^	15	20.5	3	8.1			18	18.0
Relationship status					2.71	.617		
Dating	65	89.0	21	77.8			86	86.0
Engaged	2	2.7	2	7.4			4	4.0
Married/common law	6	8.2	4	14.8			10	10.0
Gender attraction					4.58	.093		
Men only	49	67.1	12	44.4			61	61.0
Men mostly but women occasionally too	21	28.8	12	44.4			33	33.0
Men mostly but women frequently^c^	3	4.1	3	11.1			6	6.0

Abbreviations: SIAD, sexual interest/arousal disorder; TIL, thermal imaging of the labia; VPP, vaginal photoplethysmograph.

a Probable diagnosis based on *DSM-5* criteria.

b Other includes Hispanic, African, Middle Eastern, or multiple identities.

c But not more than toward men.

**Table 2:** Descriptive statistics of key variables by SIAD status and study group.^a^

	**VPP**					**TIL**				
	**Non-SIAD**		**SIAD**			**Non-SIAD**		**SIAD**		
	**Mean (SD)**	**Range**	**Mean (SD)**	**Range**	***d*** ^**b**^	**Mean (SD)**	**Range**	**Mean (SD)**	**Range**	***d*** ^**b**^
Age, y	24.5 (7.5)	18-48	23.7 (5.6)	18-37		20.8 (3.6)	18-35	21.1 (4.3)	18-35	
Relationship										
Length, mo	32.6 (47.6)	1-215	36.7 (44.4)	1-144		27.9 (28.5)	2-100	38.3 (64.9)	1.5-240	
Satisfaction	29.5 (5.0)	19-35	27.5 (5.0)	19-34		29.8 (4.1)	20-35	29.0 (4.4)	20-35	
Genital arousal, *z* score	–0.1 (0.9)	–1.9 to 2.4	0.4 (1.1)	–1.0 to 2.9		–0.1 (1.0)	–1.9 to 2.1	0.1 (1.0)	–0.9 to 3.2	
Immediate desire										
Partner	4.7 (1.7)	1-7	3.7 (2.0)	1-7	0.58	5.3 (1.6)	1-7	4.0 (1.6)	1-7	0.82
Solitary	3.0 (2.0)	1-7	2.8 (1.7)	1-6		4.4 (1.9)	1-7	3.1 (1.5)	0-5	0.70
Delayed desire										
Partner	6.1 (3.9)	0-15	3.3 (3.8)	0-12	0.72	7.1 (4.3)	0-15	3.1 (3.9)	0-15	1.00
Solitary	1.1 (1.5)	0-6	0.9 (1.5)	0-4		1.3 (2.4)	0-10	1.4 (1.7)	0-4	
Baseline desire										
Prestimulus										
Partner	1.8 (1.0)	1-5	1.7 (0.9)	1-4		1.8 (1.2)	0-5	1.1 (0.5)	0-2	0.71
Solitary	1.3 (0.6)	1-4	1.5 (0.9)	1-4		1.4 (1.1)	0-5	0.9 (0.5)	0-2	0.56
Typical (3 d)										
Partner	7.6 (4.2)	1-15	2.8 (2.8)	0-10	1.21	8.4 (4.5)	0-15	2.5 (3.5)	0-11	1.39
Solitary	1.3 (2.0)	0-10	1.0 (1.5)	0-4		2.2 (2.7)	0-9	0.7 (1.1)	0-4	0.64

Abbreviations: SIAD, sexual interest/arousal disorder; TIL, thermal imaging of the labia; VPP, vaginal photoplethysmograph.

a TIL: *n* = 23, non-SIAD; *n* = 14, SIAD. VPP: *n* = 50, non-SIAD; *n* = 13, SIAD. Women with SIAD met diagnosis criteria. Genital arousal was calculated by subtracting baseline responding from mean genital arousal to the stimulus; final genital arousal scores were standardized with *z* scores, calculated separately by study group. *P* value for significance is based on *t*-tests comparing non-SIAD vs SIAD within the study group.

b Cohen’s *d*; only presented for significant differences at (*P* < .05) comparing non-SIAD and SIAD via *t*-tests (2-sided).

### Research aim 1

**Figure 1:**
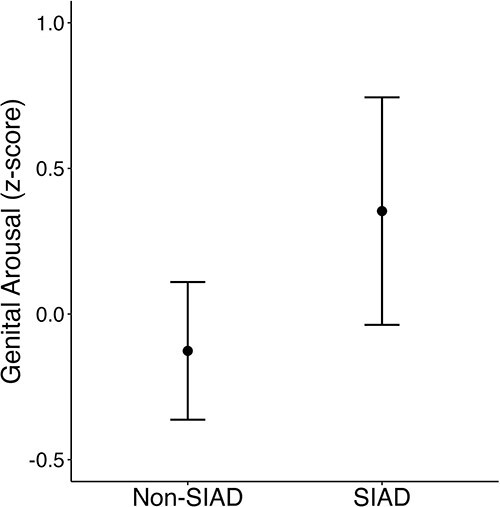
Genital arousal *z* scores predicted by SIAD status (probable diagnosis): predicted values from linear regression model. Genital arousal was calculated by subtracting baseline responding from mean genital arousal to the sexual stimulus; final genital responses were standardized with *z* scores, calculated separately by study group. *n* = 73, non-SIAD; *n* = 27 SIAD. SIAD, sexual interest/arousal disorder.

The 2-way interaction with the study group was not significant (presented in supplemental materials), indicating that results were similar across study groups. Results from the linear regression model are presented in Table 3; predicted values are presented in Figure 1. Women with SIAD exhibited significantly higher genital arousal than women without SIAD.

**Table 3:** Linear regression model: SIAD symptomology predicting genital arousal (research aim 1).^a^

**Variable**	***B* (SE)**	***P* value**
Intercept	–0.62 (0.5)	.245
Age	0.02 (0.0)	.269
Relationship length	0.00 (0.0)	.149
Gender attraction	0.06 (0.2)	.762
Study group	0.08 (0.2)	.718
SIAD status	**0.48 (0.2)**	**.042**

Abbreviations: SIAD, sexual interest/arousal disorder; TIL, thermal imaging of the labia; VPP, vaginal photoplethysmograph.

a Study group was coded as 1 = VPP and 0 = TIL. Genital arousal was calculated by subtracting baseline responding from mean genital arousal to the stimulus; final genital arousal scores were standardized with *z* scores, calculated separately by study group. Bold indicates significant focal associations at *P* < .05. *n* = 73, non-SIAD; *n* = 27, SIAD.

### Research aim 2

**Figure 2:**
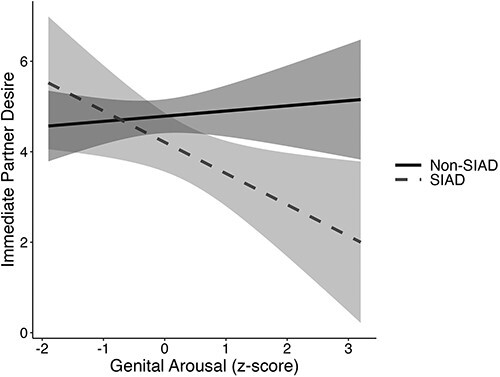
Immediate partner desire predicted by genital arousal and SIAD status: predicted values from linear regression model. Genital arousal was calculated by subtracting baseline responding from mean genital arousal to the stimulus; final genital arousal scores were standardized with *z* scores, calculated separately by study group. *n* = 73, non-SIAD; *n* = 27 SIAD. SIAD, sexual interest/arousal disorder.

The 3-way interactions with the study group were not significant (presented in supplemental materials), demonstrating that results were similar across study groups. The genital arousal × SIAD status interaction term was significant for immediate dyadic desire and indicated that increases in genital arousal predicted lower dyadic desire for women with SIAD but that genital arousal was unrelated to immediate dyadic desire for those without SIAD (Table 4). Figure 2 presents the interaction. Neither genital arousal nor SIAD status was a significant predictor for any other desire type.

**Table 4:** Linear regression model: genital arousal and SIAD status predicting sexual desire (research aim 2).^a^

	**Immediate desire**				**Delayed desire**			
	**Partner**		**Solitary**		**Partner**		**Solitary**	
**Variable**	***B* (SE)**	***P* Value**	***B* (SE)**	***P* Value**	***B* (SE)**	***P* Value**	***B* (SE)**	***P* Value**
Intercept	2.63 (0.9)	.006	0.49 (1.0)	.616	2.49 (1.9)	.199	1.24 (0.8)	.122
Age	0.03 (0.0)	.425	0.08 (0.0)	.050	–0.07 (0.1)	.299	0.00 (0.0)	.985
Relationship length	0.00 (0.0)	.730	–0.01 (0.0)	.071	0.01 (0.0)	.197	–0.01 (0.0)	.196
Gender attraction	0.62 (0.3)	.077	–0.01 (0.4)	.985	–0.13 (0.7)	.858	–1.00 (0.3)	.003
Study group	0.66 (0.3)	.058	1.41 (0.4)	<.001	0.32 (0.7)	.647	0.17 (0.3)	.593
**Desire controls**								
Prestimulus								
Partner	0.57 (0.2)	.001						
Solitary			0.83 (0.3)	.001				
Typical (3 d)								
Partner					0.68 (0.1)	<.001		
Solitary							0.50 (0.1)	<.001
SIAD status	–0.58 (0.4)	.139	–0.58 (0.4)	.172	0.29 (0.9)	.759	–0.15 (0.4)	.673
Genital arousal	0.11 (0.2)	.556	0.12 (0.2)	.581	0.27 (0.4)	.483	0.21 (0.2)	.239
SIAD × genital arousal	**–0.81 (0.4)**	**.027**	–0.27 (0.4)	.503	–0.28 (0.7)	.700	–0.02 (0.3)	.944

Abbreviations: SIAD, sexual interest/arousal disorder; TIL, thermal imaging of the labia; VPP, vaginal photoplethysmograph.

a Study group was coded as 1 = VPP and 0 = TIL. Genital arousal was calculated by subtracting baseline responding from mean genital arousal to the stimulus; final genital arousal scores were standardized with *z* scores, calculated separately by study group. Bold indicates significant focal associations at *P* < .05. *n* = 73, non-SIAD; *n* = 27, SIAD.

### Research aim 3

**Figure 3:**
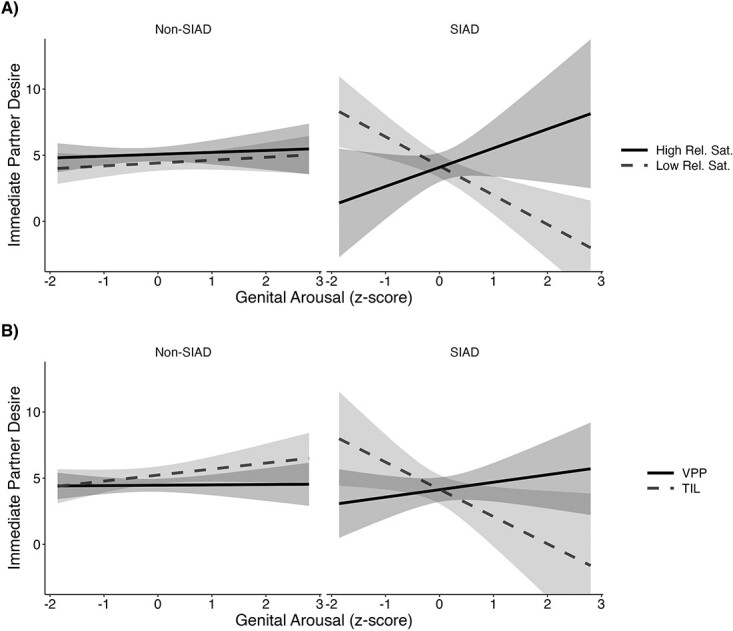
Immediate partner desire predicted by genital arousal and SIAD status, moderated by (A) relationship satisfaction and (B) study group (ie, 2 significant 3-way interactions): predicted values from linear regression model. Genital arousal was calculated by subtracting baseline responding from mean genital arousal to the stimulus; final genital arousal scores were standardized with *z* scores, calculated separately by study group. *n* = 73, non-SIAD; *n* = 27 SIAD. SIAD, sexual interest/arousal disorder.

For immediate dyadic desire, there were 2 significant 3-way interactions, which indicated that the associations among arousal, desire, and SIAD status differed by relationship satisfaction and by study group (Table 5). Interactions are presented in Figure 3. Simple slope analyses found that genital arousal was unrelated to immediate dyadic desire for women without SIAD across relationship satisfaction levels (*P* values ≥.82). For women with SIAD and high relationship satisfaction, genital arousal was positively, though not significantly, associated with immediate dyadic desire (estimate = 2.45, *P* = .060), whereas for those with low relationship satisfaction, genital arousal negatively predicted immediate dyadic desire (estimate = –1.39, *P* = .005). The interaction with the study group revealed that the negative association between genital arousal and immediate dyadic desire was significant only for the TIL group (estimate = –2.09, *P* = .029).

**Table 5:** Linear regression model: genital arousal and interactions with relationship satisfaction predicting sexual desire (research aim 3).^a^

	**Immediate desire**				**Delayed desire**			
	**Partner**		**Solitary**		**Partner**		**Solitary**	
**Variable**	***B* (SE)**	***P* value**	***B* (SE)**	***P* value**	***B* (SE)**	***P* value**	***B* (SE)**	***P* value**
Intercept	2.36 (1.0)	.026	0.93 (1.1)	.400	2.25 (2.0)	.261	1.52 (0.9)	.082
Age	0.04 (0.0)	.299	0.07 (0.0)	.126	–0.07 (0.1)	.332	–0.01 (0.0)	.857
Relationship length	0.00 (0.0)	.573	–0.01 (0.0)	.101	0.01 (0.0)	.190	–0.01 (0.0)	.080
Gender attraction	0.85 (0.4)	.027	–0.05 (0.4)	.900	0.23 (0.7)	.757	–1.05 (0.3)	.004
Study group	0.86 (0.4)	.051	1.26 (0.4)	.003	0.44 (0.7)	.536	0.00 (0.3)	.991
**Desire controls**								
Prestimulus								
Partner	0.44 (0.2)	.022						
Solitary			0.78 (0.3)	.006				
Typical (3 d)								
Partner					0.66 (0.1)	<.001		
Solitary							0.53 (0.1)	<.001
Main effects								
SIAD status	–0.36 (0.5)	.506	–0.70 (0.5)	.132	–0.08 (1.0)	.934	–0.19 (0.4)	.635
GA	0.07 (0.3)	.785	0.14 (0.2)	.551	0.52 (0.4)	.194	0.28 (0.2)	.136
RS	**0.11 (0.0)**	**.038**	0.00 (0.0)	.967	0.11 (0.1)	.234	0.05 (0.0)	.197
2-way interactions								
SIAD × GA	0.46 (0.7)	.520	–0.26 (0.6)	.656	0.23 (1.0)	.817	0.42 (0.5)	.368
SIAD × RS	–0.14 (0.1)	.175	–0.03 (0.1)	.787	–0.14 (0.2)	.408	–0.13 (0.1)	.116
GA × RS	0.00 (0.0)	.961	–0.01 (0.0)	.904	0.00 (0.1)	.994	–0.04 (0.0)	.290
Group × SIAD	–0.76 (0.9)	.387						
Group × GA	0.28 (0.4)	.503						
Group × RS	–0.14 (0.1)	.160						
3-way interactions								
SIAD × GA × RS	**0.40 (0.2)**	**.022**	0.07 (0.1)	.622	0.26 (0.2)	.286	**0.25 (0.1)**	**.036**
SIAD × GA × group	**–2.85 (1.4)**	**.045**						

Abbreviations: GA, genital arousal; RS, relationship satisfaction; SIAD, sexual interest/arousal disorder; TIL, thermal imaging of the labia; VPP, vaginal photoplethysmograph.

a Study group was coded as 1 = VPP and 0 = TIL. Genital arousal was calculated by subtracting baseline responding from mean genital arousal to the stimulus; final genital arousal scores were standardized with *z* scores, calculated separately by study group. Bold indicates significant focal associations at *P* < .05. *n* = 73, non-SIAD; *n* = 27, SIAD.

For immediate solitary and delayed partner desire, there were no significant associations with SIAD status, genital arousal, or relationship quality. For delayed solitary desire, there was a significant 3-way interaction with genital arousal, SIAD status, and relationship quality. However, simple slope analyses showed that individual slopes were not significant at *P* < .05, although there were marginal positive associations for women without SIAD who had low relationship satisfaction and for women with SIAD who had high relationship satisfaction (*P* values <.06).

## Discussion

The goal of the current study was to assess the role of SIAD status and relationship quality in the associations between genital arousal and responsive desire. The linear regression model demonstrated a stronger genital response among women with a probable SIAD diagnosis. This surprising finding stands in contrast to previous studies that documented either no differences or lower arousal among women with various sexual difficulties as compared with those with healthy sexual functioning.^7^^,^^36–39^ Our contrasting findings could be due to differing definitions; that is, each of these previous studies used a different categorization of sexual dysfunction. The present work is the first known study to use the diagnostic criteria for SIAD, the new combined diagnosis for female sexual dysfunctions involving arousal and desire inthe most recent update to the *DSM* (5th edition).^4^ The updated diagnosis combines arousal and desire difficulties, largely due to their high comorbidity,^40^^,^^41^ and could capture a cluster of symptoms that may have a unique presentation of genital response in the laboratory. It also introduces diagnosis group heterogeneity. The few previous studies documenting decreased genital arousal among women with sexual dysfunctions found the decreases for those who self-reported difficulties with genital arousal.^10^^,^^37^ It is likely that SIAD status is unassociated with problems in genital arousal when tested in a controlled laboratory experiment.

The associations between genital arousal and responsive desire were significant only for women with SIAD and depended on relationship satisfaction and desire type. Regarding the immediate dyadic desire of women with SIAD, relationship satisfaction moderated the link between genital arousal and desire. For those with low relationship satisfaction, there was a negative association: as genital arousal increased, immediate desire for sex with a partner decreased among women with SIAD. It is possible that experiences of high genital arousal could trigger an avoidance response for those in low-quality relationships who find sex with a partner unsatisfying or otherwise negative (physically or emotionally).

For women affected by SIAD who have high relationship satisfaction, genital arousal was either positively associated with immediate partner desire (VPP group) or unassociated with it (TIL group). In contrast, no associations were found for immediate solitary or delayed partner desire. The differences across desire measures support recent calls for researchers to carefully consider desire targets when examining sexual desire, as they likely represent distinct sexual experiences with distinct incentive values.^19^

The current findings indicate that women affected by SIAD may experience desire that is more strongly connected to their physical sexual response. This may result from processing their genital responses as signals of potential sexual activity with their partners, which may be positive or negative depending on their relationship context. That this association between genital response and desire was not found for women without SIAD, despite strong connections between subjective sexual arousal and partner desire,^18^ suggests that genital arousal may be a stronger trigger of sexual motivation or aversion for women with SIAD and that relationship satisfaction plays a significant role in how their genital arousal translates to sexual desire.

The stimulus used in the current study broadly depicted the same sexual activities as the previous studies: a man and a woman engaging in penile-vaginal intercourse and oral sex (cunnilingus and fellatio). The models were physically attractive, which can positively affect genital arousal to sexual stimuli.^42–44^ Our film also depicted mutually pleasurable and affectionate sexual activity. Emotional intimacy is a key motivator of sexual activity^16^^,^^17^ and may be particularly motivating for women with arousal and/or desire disorders or for whom sexual intercourse may offer little direct physical pleasure. Depictions of penile-vaginal intercourse may be less arousing than activities more directly connected to women’s pleasure or to lack of penetrative pain, such as cunnilingus.^38^ Following the incentive motivation model,^1^ stimuli depicting intimate and affectionate penile-vaginal intercourse and cunnilingus among attractive models, as in the current study, could be more strongly incentivized for women with arousal and desire challenges, which could explain their relatively higher responses.

### Limitations

Limitations include the relatively small proportion of women with SIAD as compared with women without SIAD and the differences in genital measures across study groups. Our screener was based on self-report and was not a clinical diagnosis of SIAD, which limits clinical implications and accounts for our use of “probable SIAD diagnosis” and “SIAD symptoms” throughout this article; nonetheless, the screener mirrored exactly the SIAD criteria in the *DSM-5* and was verified through a clinical interview conducted by trained researchers. The homogeneity of the sample and the requirement to attend in-person laboratory session reduce generalizability to more diverse populations—a critical direction for future research.

## Conclusion

As a group, women affected by SIAD do not exhibit reduced genital responses to sexual stimuli in the laboratory and may actually exhibit stronger arousal responses with sufficiently incentivized sexual stimuli vs healthy controls. The connection between genital arousal and responsive desire may be stronger and more variable among women with SIAD than women without it (who exhibited no associations), and the association may depend on relationship satisfaction and desire type. Links between genital arousal and responsive partner desire among women with SIAD were suggestive of an avoidance model in response to heightened genital arousal unless relationship satisfaction was high. The findings have clinical implications for the potential role of attending to genital arousal sensations as a means of triggering sexual desire for women with SIAD who are satisfied in their relationships. More broadly, the findings emphasize the importance of individual treatment plans based on patients’ current circumstances and presenting symptoms. They could also inform the use of specific treatments, such as mindfulness-based interventions^5^^,^^45^ or cognitive behavioral therapy,^46^ which can enhance self-awareness of bodily experiences and thought patterns surrounding sexual response, how they interact with feelings about the relationship, and how these may translate to sexual motivation within the relationship. Clinicians and researchers should consider the evolving nature of diagnosing female sexual dysfunctions and the resulting implications for accurately assessing and addressing female sexual health.

## Funding

Funding for this study was from an Operating Grant (MOP-130347) from the Canadian Institutes of Health Research awarded to M.L.C. and L.A.B. The funding agency had no role in study design, data collection and analysis, decision to publish, or preparation of the manuscript.

## Conflicts of interest

None declared.

## Data availability

Data analyzed in the current study may be requested by contacting Dr Meredith L. Chivers at mchivers@queensu.ca.

## Author contributions

Shari M. Blumenstock (Conceptualization [lead], Formal analysis [lead], Investigation [lead], Visualization [lead], Writing—original draft [lead], Writing—review & editing [lead]), Kelly Suschinsky (Conceptualization [equal], Data curation [lead], Methodology [lead], Project administration [lead], Writing—review & editing [supporting]), Lori A. Brotto (Conceptualization [supporting], Data curation [supporting], Funding acquisition [equal], Methodology [supporting], Writing—review & editing [supporting]), Meredith L. Chivers (Conceptualization [supporting], Data curation [equal], Formal analysis [supporting], Funding acquisition [equal], Methodology [supporting], Project administration [supporting], Supervision [supporting], Writing—original draft [supporting], Writing—review & editing [supporting]).

## Supplementary Material

ADesSIAD_Supp_qdae036
