# Supplementary material for: Genital arousal and responsive desire among women with and without sexual interest/arousal disorder symptoms
Source: J Sex Med. 2024 Apr 6;21(6):539–47. doi: 10.1093/jsxmed/qdae036 (PMC11144479; doi:10.1093/jsxmed/qdae036)
Supplement: ADesSIAD_Supp_qdae036 [file adessiad_supp_qdae036.pdf]

Supplemental Materials for
"Genital Arousal and Responsive Desire Among Women With and Without Sexual Interest/Arousal Disorder Symptoms"

Linear Regression Model Results: SIAD Symptomology Predicting Genital Arousal (RQ1)

Variable	*B (SE)*	*p*
Intercept	-0.64(0.5)	.233
Age	0.02(0.0)	.269
Relationship length	0.00(0.0)	.153
Gender attraction	0.07(0.2)	.747
Study group	0.13(0.3)	.620
SIAD status	0.55(0.3)	.072
SIAD x Group	-0.17(0.5)	.153

Linear Regression Model Results: Genital Arousal and SIAD Status Predicting Sexual Desire (RQ2)

	Imm Partner	Imm Solitary	Del Partner	Del Solitary
Variable	B(SE)	B(SE)	B(SE)	B(SE)
Intercept	2.42 (0.96) *	0.49 (1.00)	2.41 (1.98)	1.35 (0.82)
Age	0.04 (0.04)	0.08 (0.04) +	−0.07 (0.07)	0.00 (0.03)
Relationship length	0.00 (0.00)	−0.01 (0.01) +	0.01 (0.01)	−0.01 (0.00)
Gender attraction	0.62 (0.35) +	0.02 (0.38)	−0.11 (0.71)	−1.02 (0.33) **
Study group	0.81 (0.41) +	1.61 (0.45) ***	0.39 (0.83)	−0.10 (0.38)
Desire Controls				
Pre-stimulus partner	0.57 (0.17) **			
Pre-stimulus solitary		0.77 (0.26) **		
Baseline 3-day partner			0.68 (0.09) ***	
Baseline 3-day solitary				0.52 (0.07) ***
SIAD status	−0.41 (0.50)	−0.22 (0.56)	0.47 (1.13)	−0.47 (0.48)
Genital arousal (GA)	−0.05 (0.25)	0.11 (0.28)	0.15 (0.51)	0.18 (0.23)
SIAD x GA	−0.67 (0.47)	−0.43 (0.53)	−0.41 (0.95)	−0.14 (0.43)
GA x Group	0.42 (0.40)	0.04 (0.44)	0.33 (0.81)	0.08 (0.37)
SIAD x Group	−0.37 (0.76)	−0.82 (0.85)	−0.36 (1.54)	0.80 (0.70)
GA x SIAD x Group	−0.35 (0.72)	0.31 (0.80)	0.31 (1.46)	0.33 (0.67)

Note. Study group was coded as 1 = Vaginal photoplethysmography (VPP), 0 = Thermal imaging (TIL). Genital arousal calculated by subtracting baseline responding from mean genital arousal to the stimulus; final genital arousal scores were standardized using z-scores, calculated separately by study group. Relationship satisfaction and length were mean-centered. Bold font indicates significant focal associations at $p < .05$. N = 73 non-SIAD women and 27 SIAD women.
*** p < .001. ** p< .01. * p < .05. + p < .10.

Linear Regression Model Results: Genital Arousal & Interactions with Relationship Satisfaction Predicting Sexual Desire (RQ3)

	Imm Partner	Imm Solitary	Del Partner	Del Solitary
Variable	B(SE)	B(SE)	B(SE)	B(SE)
Intercept	2.36 (1.04) *	1.02 (1.16)	2.23 (2.11)	1.67 (0.91) +
Age	0.04 (0.04)	0.07 (0.04)	−0.07 (0.08)	−0.01 (0.04)
Relationship length	0.00 (0.01)	−0.01 (0.01) +	0.02 (0.01)	−0.01 (0.01) +
Gender attraction	0.85 (0.38) *	−0.08 (0.43)	0.32 (0.78)	−1.14 (0.36) **
Study group (Group)	0.86 (0.43) +	1.74 (0.50) ***	0.24 (0.88)	−0.22 (0.41)
Desire Controls				
Pre-stimulus partner	0.44 (0.19) *			
Pre-stimulus solitary		0.58 (0.31) +		
Baseline 3-day partner			0.67 (0.09) ***	
Baseline 3-day solitary				0.53 (0.08) ***
Main effects				
SIAD status	−0.36 (0.54)	−0.22 (0.62)	−0.24 (1.22)	−0.55 (0.52)
Genital arousal (GA)	0.07 (0.27)	0.18 (0.31)	0.44 (0.54)	0.27 (0.25)
Relationship satisfaction(RelSat)	0.11 (0.05) *	0.04 (0.06)	0.07 (0.11)	0.05 (0.05)
2-way interactions				
SIAD x GA	0.46 (0.71)	0.04 (0.82)	0.10 (1.46)	0.06 (0.67)
SIAD x RelSat	−0.14 (0.10)	−0.07 (0.12)	−0.10 (0.21)	−0.13 (0.10)
GA x RelSat	0.00 (0.05)	−0.02 (0.06)	0.00 (0.10)	−0.04 (0.05)
Group x SIAD	−0.76 (0.88)	−1.23 (1.01)	−0.06 (1.76)	1.17 (0.81)
Group x GA	0.28 (0.42)	−0.11 (0.48)	0.25 (0.85)	0.01 (0.39)
Group x RelSat	−0.14 (0.10)	−0.18 (0.12)	0.12 (0.20)	0.01 (0.09)
3-way interactions				
SIAD x GA x RelSat	0.40 (0.17) *	0.21 (0.19)	0.18 (0.34)	0.15 (0.16)
SIAD x GA x Group	−2.85 (1.40) *	−0.63 (1.61)	−1.51 (2.83)	1.97 (1.29)
SIAD x RelSat x Group	0.23 (0.20)	0.23 (0.23)	−0.29 (0.40)	0.03 (0.19)
GA x RelSat x Group	0.01 (0.09)	0.10 (0.10)	−0.01 (0.17)	0.00 (0.08)
4-way interaction				
SIAD x GA x RelSat x Group	−0.13 (0.48)	−0.35 (0.55)	0.96 (0.96)	−0.36 (0.45)

Note. Study group was coded as 1 = Vaginal photoplethysmography (VPP), 0 = Thermal imaging (TIL). Genital arousal calculated by subtracting baseline responding from mean genital arousal to the stimulus; final genital arousal scores were standardized using z-scores, calculated separately by study group. Relationship satisfaction and length were mean-centered. Bold font indicates significant focal associations at $p < .05$. N = 73 non-SIAD women and 27 SIAD women.
*** p < .001. ** p< .01. * p < .05. + p < .10.
